# Exploring Lifestyle Habits, Physical Activity, Anxiety and Basic Psychological Needs in a Sample of Portuguese Adults during COVID-19

**DOI:** 10.3390/ijerph17124360

**Published:** 2020-06-18

**Authors:** Raul Antunes, Roberta Frontini, Nuno Amaro, Rogério Salvador, Rui Matos, Pedro Morouço, Ricardo Rebelo-Gonçalves

**Affiliations:** 1 Department of Human Kinetics, Polytechnic Institute of Leiria, 2411 Leiria, Portugal; nuno.amaro@ipleiria.pt (N.A.); rogerio.salvador@ipleiria.pt (R.S.); rui.matos@ipleiria.pt (R.M.); pedro.morouco@ipleiria.pt (P.M.); ricardo.r.goncalves@ipleiria.pt (R.R.-G.); 2 Life Quality Research Centre (CIEQV), Polytechnic Institute of Leiria, 2411 Leiria, Portugal; 3 Sport Science School of Rio Maior (ESDRM), Polytechnic Institute of Santarém, 2040 Santarem, Portugal; 4 Center for Health Technology and Services Research (CINTESIS.UA), School of Health Sciences, University of Aveiro, (Campus Universitário de Santiago), 3810 Aveiro, Portugal; roberta_frontini@hotmail.com; 5 Center for Innovative Care and Health Technology (ciTechCare), Polytechnic of Leiria, 2411 Leiria, Portugal; 6 CIPER, Faculdade de Motricidade Humana, Universidade de Lisboa, 1495 Cruz Quebrada Dafundo, 1649004 Lisbon, Portugal; 7 Research Unit for Sport and Physical Activity, University of Coimbra, 3040 Coimbra, Portugal

**Keywords:** Coronavirus, exercise, healthy eating, sleep routines, anxiety, basic psychological needs

## Abstract

This cross-sectional study aimed to characterize the lifestyle habits, anxiety levels and basic psychological needs (BPN), in Portuguese adults during the Coronavirus Disease 2019 (COVID-19) pandemic, including a comparison between genders and age groups. In total, 1404 adults (36.4 ± 11.7 years; 69.6% female) answered sociodemographic data and three instruments: the International Physical Activity Questionnaire, the Basic Need General Satisfaction Scale and the State-Trait Anxiety Inventory. Males revealed higher values for the total energy expenditure (z = −2.26; *p* = 0.024; $\eta^{2}$ = 0.004) and for the level of satisfaction of competence (z = −2.62; *p* = 0.009; $\eta^{2}$ = 0.005). Females showed higher scores for the anxiety state (z = −7.87; *p* ≤ 0.001; $\eta^{2}$ = 0.044) and anxiety trait (z = −6.49; *p* ≤ 0.001; $\eta^{2}$ = 0.030). Regarding age, higher values for the anxiety trait (*p* ≤ 0.001; $\eta_{H}^{2}$ = 0.030) were found in the 18–34 years-old group compared to all the other age groups, also presenting significantly higher values of total energy expenditure (χ² = 13.93; *p* = 0.008; $\eta_{H}^{2}$ = 0.007) when compared to the 35–44 years-old group. Significant differences were observed between the 18–34 years-old group and the other age groups for the satisfaction of competence (χ² = 40.97; *p* ≤ 0.001; $\eta_{H}^{2}$ = 0.026), except for the >65 years-old group. Strategies for promoting well-being during periods of social isolation should consider the role of psychological dimensions and lifestyle habits according to the gender or age group.

## 1. Introduction

The new Coronavirus Disease 2019 (COVID-19) was first identified in the Chinese city of Wuhan in December 2019. It was declared by the World Health Organization [1] as a public health emergency of international concern on 30 January 2020. Later, on 11 April 2020, it was declared a pandemic [2].

Considering that this virus spreads out worldwide among humans at an exponential rate, several countries have adopted prevention measures. These measures included the restriction of the free movement and circulation of people [3] such as quarantine, which, however, should only be applied as part of a more comprehensive package of measures [4]. It is important to define the concept of quarantine, which represents the restriction of activities or the separation of a non-sick person after contact with an infected individual to monitor his or her symptoms and detect earlier cases. Instead, physical isolation represents the separation of infected people from the rest of the population, aiming to prevent the spread of the infection [4]. On 18 March, a state of emergency was decreed in Portugal, which implemented a set “of extraordinary and urgent measures to restrict rights and freedoms, especially with regard to the rights of free movement and economic freedoms, in articulation with European authorities to prevent the virus transmission” (Decree of the President of the Republic no. 14-A/2020).

Although quarantine and physical isolation measures may have a positive effect in protecting peoples’ physical health by preventing and mitigating the virus transmission, the application of these restraint measures can have a long-lasting and wide-ranging negative psychosocial impact [5,6,7]. Some studies have shown several negative psychological effects of social isolation, such as high levels of anxiety, stress, fear, or even the presence of depressive symptoms that can persist beyond that period [5,8].

Positive social interactions may be considered as basic human needs, causing social cravings similar to food cravings [9]. Psychological well-being can be predicted by the degree of satisfaction of three basic psychological needs, autonomy (i.e., the subject’s ability to regulate his or her own actions), competence (i.e., the subject’s efficiency in interacting with the environment) and relatedness (i.e., the subject’s ability to search for and develop connections and interpersonal relationships), according to the theory of basic psychological needs [10]. Thus, physical isolation and quarantine may come into conflict with basic psychological needs by keeping people away from each other. This may inhibit individuals from seeking and developing interpersonal connections and relationships as well as hinder the regulation of their own actions.

Considering the possible consequences that this period of social isolation may have on the levels of stress and anxiety, the WHO emitted a set of deliberations to minimize its effects. Among other measures, the need to restrict the time spent watching COVID-19 news and to seek reliable information in official media was highlighted, along with the need to maintain family routines and to seek a healthy lifestyle, namely through regular physical activity, healthy eating and regular sleep routines [11]. The practice of physical activity has a fundamental role, particularly considering that changes in daily routines may lead to an increase in sedentary behaviors and contribute to higher anxiety levels [12]. In fact, physical activity not only has unquestionable benefits for physical health, but it is also associated with a reduction in anxiety levels [13,14], even when there is a replacement of outdoor activities with home-based activities [15]. After the President of the Republic decreed the state of emergency, the government dictated a set of mandatory rules. It was possible, in Portugal during this time, to make short trips with the purpose to practice physical activities. However, the exercise of collective physical activities was prohibited, and gyms and sports clubs were closed.

Although previous studies [16,17] have demonstrated the impact of physical isolation on people’s physical and mental health, this pandemic and its consequences are very recent. Moreover, it would be of the utmost importance to understand the role of gender, especially considering that policy responses had not yet addressed the gendered impacts of disease outbreaks [18]. Furthermore, along with gender differences, research should also consider the specificities of age differences, especially considering that COVID-19 has a different impact on different age-groups [19]. Thus, we intended to explore the role of gender and age in lifestyle habits, physical activity, anxiety and basic psychological needs. To the best of our knowledge, there is as of yet no published paper characterizing these variables in the Portuguese population. Bearing in mind the concerns of a multitude of health entities worldwide, and the fact that new waves of the virus are expected, it is extremely important to understand people’s life habits during this period. The aim of this study was twofold: (1) to better understand the lifestyle habits of the Portuguese adult population during the COVID-19 pandemic, namely regarding sleeping habits, eating habits and physical activity habits; and (2) to explore the anxiety levels and the satisfaction of basic psychological needs during the COVID-19 pandemic in Portuguese adults. Moreover, we consider it essential to study variables such as the anxiety levels and the satisfaction of basic psychological needs. Our goal is to be able to contribute, with our results, to helping future interventions to be more targeted, so as to generate important trails that will help identify possible fragile groups (e.g., identification of age groups that need more attention during future waves).

## 2. Materials and Methods

### 2.1. Study Design and Procedures

This cross-sectional study was conducted in the period between 1–15 April, during which a state of emergency was decreed by the Portuguese president. This survey involving community adults included a number of self-reported domains of an individual’s behavior and feelings towards the confinement period of COVID-19.

A Google form was used as the survey platform for electronic distribution, while social media and newspapers were used to advertize and recruit possible volunteers. The volunteers received no compensation for their participation. Each questionnaire assessed four domains: sociodemographic data, physical activity levels, anxiety-state and anxiety-trait levels, and basic psychological needs satisfaction measurements. The survey with sociodemographic questions was specifically developed for this purpose and reviewed by four experts in exercise and psychology. The other assessment domains comprised validated instruments for the Portuguese population. Respondents took an average time of 14 min to complete the survey. Procedures followed standards for research in sports medicine and were performed according to the Declaration of Helsinki.

### 2.2. Participants

Subjects were recruited by convenience sample method by assembling a group of individuals who volunteered to participate and were only eligible if they were aged over 18 years old and had the Portuguese nationality. The sample was composed of a total of 1404 respondents (36.4 ± 11.7 years of age), ranging from 18 to 89 years of age, of which 977 (69.6%) were women (35.7 ± 11.6 years old), 426 (30.3%) were men (38.1 ± 11.6 years old) and only one respondent preferred not to specify (35.0 years old; 0.1%). Respondents were fully informed regarding the nature of the study, the procedures for data recording and the voluntary nature of their participation. They were also informed that they could withdraw from the study at any time. Subjects provided their consent before the survey’s completion, and anonymity was guaranteed.

### 2.3. Variables

In the sociodemographic survey, respondents were invited to answer simple questions regarding age, gender, marital status, living status during confinement and academic level. Moreover, they were asked to self-report about sleep duration (inferred from regular bedtime and getting up) and quality of the sleep, the amount and frequency of food intake, and the time spent watching pandemic-related news, by answering the following questions (regarding the present moment, i.e., during the COVID-19 pandemic):

- Usual sleep duration: Do you go to bed and get up at about the same time you used to do?
- Sleep quality satisfaction: Are you satisfied with the quality of your sleep?
- Higher food frequency: Do you feel you eat more often than usual?
- Higher food quantity: Do you feel you eat more than usual?
- Careful food selection: Are you more careful with food selection than usual?

In the previous questions, participants should answer yes or no.

- Time spent watching, reading or listening to the news about Coronavirus: During COVID-19, how much time do you spend watching, reading, or listening to the news about Coronavirus? Participants should answer none, less than 1 h, 1–3 h, more 3 h and another option.

To assess physical activity, the International Physical Activity Questionnaire (IPAQ – short form) was applied. It is widely used to estimate the frequency and duration of low, moderate and high physical activity levels and has been validated for 12 countries, including Portugal [20]. This questionnaire includes four questions regarding the specific types of physical activity, e.g., walking and moderate and vigorous activities, in terms of the frequency and duration of each specific type of activity, as well as the time spent seated per day in a week. Data are then converted into MET-min/week (metabolic equivalent) by computing the marked minutes per week in each category of activities by their specific metabolic equivalent.

The assessment of the anxiety trait and anxiety state was provided by the Portuguese version [21] of the State-Trait Anxiety Inventory (STAI trait, STAI state) [22]. This questionnaire is composed of two blocks (Form 1 and Form 2) of 20 statements each, evaluated in a four-point Likert scale. Form 1—STAI State—evaluates transient or temporary anxiety, i.e., the anxiety the person is feeling at the present moment. Form 2—STAI Trait—assesses the dispositional or general anxiety. Examples of items include: “I’m worried” (state) and “I often feel that I´m not able” (trait). Internal consistency in this study proved to be good (state α = 0.93; trait α = 0.93).

The Portuguese version [23] of the Basic Need General Satisfaction Scale (BNSG-S) [24] was also used. This questionnaire comprises 21 items, to which the subjects respond on a seven-point “Likert” scale ranging between one (“totally disagree”) to seven (“totally agree”). The items are grouped into three factors assessing the satisfaction with three types of needs (autonomy: seven items; competence: six items; and relatedness: eight items). Examples of items include: “I feel like I can decide for myself how to live my life” (autonomy), “I really like the people I interact with” (relatedness) and “I often do not feel very capable” (competence, reversed item). Internal consistency for the subscales, in this study, ranged from acceptable to good (autonomy α = 0.65; competence α = 0.78; and relatedness α = 0.66).

### 2.4. Data Analysis

The counts (and proportions), means, standard deviations (sd) and 95% confidence interval (95% CI), and medians (interquartile range, IQR) were calculated to describe the categorical and continuous variables for the total sample. Normality was checked using the Shapiro–Wilk test and by visual inspection of normality plots. Since the assumptions of a normal distribution were violated for all variables (*p* ≤ 0.001), non-parametric statistics were preferably used in the entire analysis.

Gender-specific statistics were calculated for sleep (usual sleep duration and sleep quality satisfaction); food frequency and quantity of food; careful food selection; time spent watching, reading or listening to the news; total METS; anxiety state and anxiety trait; and autonomy, competence and relatedness. Mann–Whitney U and Pearson’s Chi-square tests were used to test the effect of gender on the abovementioned variables, for continuous and categorical variables, respectively. For the gender-related analysis, one respondent was excluded. Participants’ age group was classified as follows: 18–34 years old, 35–44 years old, 45–54 years old, 55–64 and >65 years old. The age-related variation was computed using the Kruskal–Wallis and Pearson’s Chi-square tests on sociodemographic, physical activity, anxiety and basic psychological needs satisfaction variables, for continuous and categorical variables. In the categorical variables, the standardized adjusted residuals were reported for each group to interpret the significant differences.

The reported estimate of the effect size measures followed the recommendations [25] for non-parametric tests. Those estimates assumed values from 0 to 1, and multiplied by 100 they indicate the percentage of variance in the dependent variable explained by the independent variable. The effect statistics were interpreted following the proposed magnitude thresholds. The correlation coefficients were interpreted using the proposed thresholds by Hopkins and colleagues [26]: trivial (r < 0.1), small (0.1 < r < 0.3), moderate (0.3 < r < 0.5), large (0.5 < r < 0.7), very large (0.7 < r < 0.9) and nearly perfect (r ≥ 0.9). Significance was set at 5%. All data analyses were computed using the IBM Statistical Package for Social Science software for Windows (SPSS v.26.0, IBM Corp., Armonk, NY, USA).

## 3. Results

The sample characteristics are presented in Table 1. It is worth noting that 447 respondents (31.8%) were classified in the lower physical activity category, 697 subjects (49.6%) were classified as moderate, while only 260 of the participants (18.5%) were categorized as engaging in high physical activity.

Gender was a consistent source of variation (Table 2), particularly for sociodemographic data such as age (*p* = 0.001; $\eta^{2}$ = 0.009), sleep quality satisfaction (*p* ≤ 0.001; ϕ = 0.127), higher food frequency (*p* = 0.018; ϕ = -0.063) and quantity (*p* = 0.001; ϕ = −0.085). The standardized adjusted residuals for the sleep quality satisfaction variable varied between a 4.8 and a maximum of −4.8 (male) and between −4.7 and 4.7 (female); for the higher food frequency variable, they ranged between −2.4 and 2.4 (male) and between 2.3 and −2.3 (female); for the food quantity variable, they varied between a minimum of −3.2 and a maximum of 3.2 (male) and between 3.1 and −3.1 (female). Male subjects reported significant higher values for the total energy expenditure (*p* = 0.024; $\eta^{2}$ = 0.004) and competence (*p* = 0.009; $\eta^{2}$ = 0.005), while female participants revealed higher results for the anxiety state (*p* ≤ 0.001; $\eta^{2}$ = 0.044) and anxiety trait (*p* ≤ 0.001; $\eta^{2}$ = 0.030).

The comparisons among age-groups (Table 3) revealed significant differences for the usual sleep duration (*p* ≤ 0.001; ϕ = 0.175), careful food selection (*p* = 0.002; ϕ = 0.112) and time spent watching, reading or listening to news (*p* ≤ 0.001; ϕ = 0.126). The standardized adjusted residuals in the usual sleep duration variable varied between: −6.3–6.3 (18–34 years), −2.7–2.7 (35–44 years), −3.0–3.0 (45–54 years), −2.9–2.9 (55–64 years) and −0.7–0.7 (>65 years). For the careful food selection, the standardized adjusted residuals ranged between: −3.7–3.7 (18–34 years), −0.7–0.7 (35–44 years), −3.1–3.1 (45–54 years), −1.3–1.3 (55–64 years) and −0.5–0.5 (>65 years). Lastly, for the variable time spent watching, reading or listening to the news about Coronavirus, the standardized adjusted residuals varied between: −4.2–3.7 (18–34 years), −1.1–1.2 (35–44 years), −5.0–4.3 (45–54 years) and −1.7–2.1 (>65 years).

The comparisons also revealed significant differences for the total energy expenditure (*p* = 0.010; $\eta_{H}^{2}$ = 0.007), anxiety trait (*p* ≤ 0.001; $\eta_{H}^{2}$ = 0.029) and competence (*p* ≤ 0.001; $\eta_{H}^{2}$ = 0.026). The post-hoc analysis showed significant differences between the youngest group and all remaining groups for the anxiety trait. In fact, the 18–34 years-old group presented significantly lower values when compared to the other groups for competence, except for the older group. The 18–34 years-old group presented significantly higher values for the total energy expenditure when compared to the 35–44 years-old group.

## 4. Discussion

This study had two main purposes: (1) to characterize the lifestyle habits of a sample of the Portuguese adult population during the COVID-19 pandemic, particularly by analyzing sleep, eating and physical activity habits; and (2) to explore the anxiety levels and the satisfaction with basic psychological needs during this particular period.

Considering sleeping habits, most participants reported maintaining routine habits, particularly the number of hours of sleep. On the contrary, a recent study carried out in China [27] during the COVID-19 pandemic found a high prevalence of poor sleep quality in the population. Our results also showed altered eating habits, since a significant percentage of participants reported that they started to eat more often (45.2%), in larger quantities (31.6%) and that they had no careful food selection (58.1%). These results are in line with some studies that showed that in situations of greater stress and anxiety people tend to regulate their emotions through food [28,29]. However, a better food selection reported by 41.9% of the respondents may be due to the fact that some people spend more time at home, some of whom may have more time to cook, meeting the recommendations that have been given by health organizations [4].

As regards the practice of physical activity and exercise, our results reveled that most of the participants engaged in regular physical activity (being classified in the moderate and high categories of the IPAQ). These results are particularly important considering that the literature has acknowledged the positive impact of physical activity on mental health [13], particularly during social isolation and quarantine [12]. A recent work reported that higher levels of physical activity were associated with lower levels of anxiety during COVID-19 [30]. Research has further reinforced that physical activity has played an important role in physical health during COVID-19 [31,32]. Furthermore, our results revealed gender-related differences, with men showing significantly higher levels of physical activity compared to women. An age difference was also found, with younger adults (18–34 years) showing lower levels of physical activity. Understanding the levels of physical activity and exercise during COVID-19 is of the utmost importance and can contribute to the anticipation and/or reinforcement of physical activity promotion strategies for possible second and third waves of this pandemic or even in similar situations where social isolation is mandatory.

It is important to emphasize that the anxiety state levels were higher than the anxiety trait ones, something particularly relevant in this pandemic period. Furthermore, in our study, women presented higher levels of state anxiety and trait anxiety when compared to men. These results are in line with past investigations [33], suggesting that these differences may remain even during a pandemic situation and subsequent social confinement. An age-related variation was also found, with the youngest participants (18–34 years) showing higher levels of trait anxiety, which is consistent with previous research [34]. Thus, regarding anxiety levels, the results of this study suggest that women and younger adults may be two groups with greater vulnerability, which is in line with previous studies. In fact, in a recent investigation [27], the authors suggested that younger individuals may spend a great deal of time thinking and worrying about the outbreak, which may explain the higher levels of anxiety symptoms. This may be a possible explanation for the higher levels of anxiety levels found in our study.

Regarding the satisfaction of basic psychological needs, our results showed statistically significant differences among community adults. The need for competence seems to be influenced by both participants’ age and gender, being significantly lower in women and in the younger elements of our sample. These results seem to contradict previous studies [35,36], which point out the absence of gender differences in the experience of satisfying the three basic needs. In fact, in our study, males and females differed in their needs for autonomy and relatedness. The exceptional situation experienced in this period may contribute to a lower perception of satisfaction of competence, particularly in the female gender, mostly due to changes in the usual routine, namely regarding activities that may provide greater interaction with the environment and that may help experience a better satisfaction of this psychological basic need.

On the other hand, the age-related variation taking into account the need for competence showed statistically significant differences, with the youngest adults (18 to 34 years old) reporting lower values of satisfaction. These results seem to contradict other studies [37], which point out that there are no age differences in the experience of satisfaction of basic psychological needs. Still, it is necessary to consider whether these differences in the perception of competence may refer to the subject’s capacity for effectiveness in interacting with the environment [38]. Our results suggest the possibility that younger adults were the ones that experienced greater self-reported changes in their daily routines during this period of COVID-19, as a result of contingency measures (e.g., changes in school/university routines, work, hobbies, etc.) in opposition to older adult, who may not even be working (e.g., retired). Staying at home can lead to higher levels of stress, anxiety and mental distress. Moreover, the situation of confinement may cause people to practice less physical activity since they are no longer able to go to gyms or sports clubs. This may be significantly alarming considering that Portugal is one of the countries with the lowest rates of physical activity in normal life situations [39]. However, during a social isolation period where it is not possible to perform some of the usual outdoor activities, home-based activities gain an additional relevance. These activities may be bodyweight training, dance-based aerobic exercise and, if possible, aerobic high-intensity exercise using stationary bikes or rowing ergometers, along with self-paced protocols or even domestic chores. These activities can be combined with stretching and active gamming and should, if possible, be supervised by professionals (e.g., exercise physiologists).

Our data highlighted the importance of examining the implications of changing routines for meeting the satisfaction of these three basic needs (in both genders and different age groups), which is emphasized in periods like the ones we are now living. Psychological basic needs play a fundamental role in an individual’s daily life, being essential conditions for people’s optimal functioning and for their personal well-being, as has been discussed in past literature [40]. Despite the observed differences in the present study, it should be noted that the effect sizes ranged between trivial and small. One should remain cautious about generalizing our results, since these may not be representative of the Portuguese population. The cross-sectional design of our study does not allow causal inferences to be made. Future studies should also seek to identify and better understand factors that may be related to higher levels of state anxiety in this period of confinement, namely those related to lifestyle habits (including an analysis of the relationship between levels of physical activity, NPB satisfaction and anxiety).

## 5. Conclusions

As a period of social confinement is necessary for the protection of community health (by helping to reduce the risk of contamination), it is important to understand people’s lifestyle habits (e.g., food, sleep, physical activity) during this time. Moreover, it is crucial to explore some psychological dimensions that play a significant role in people’s quality of life and well-being (e.g., anxiety and basic psychological needs). The definition and effectiveness of intervention strategies for promoting the quality of life and well-being during periods of social isolation could be enhanced by considering the role of psychological dimensions and lifestyle habits according to the gender or age group.

In fact, the design of prevention and intervention programs should consider, on the one hand, some of these variables, namely the effect of gender and age. On the other hand, the results of the present study may contribute to creating strategies for an improved dissemination of the message that should be spread by the competent entities (specifically, health organizations), particularly when promoting mental health. For instance, our results emphasize the importance of continuing to spread the message of getting physically active during this pandemic, specifically taking into account that the interaction with the environment is clearly limited during COVID-19. Moreover, our results suggest the importance of working towards creating strategies to promote healthy eating habits, by not eating more or more often and by carefully choosing what to eat. Our results highlight the importance of identifying which groups may face more difficulties adopting healthy behaviors (e.g., physical activity, healthy food choices and sleep routines). By identifying these vulnerable groups, intervention strategies may be more targeted, and the effectiveness of health strategies may be improved. In fact and according to our results, women and younger adults may need greater attention, firstly because they appear to practice less physical activity and secondly in view of the presence of higher levels of anxiety.

## Figures and Tables

**Table 1:** Summary of the descriptive statistics for the sample characteristics (*n* = 1404).

	*n* (%)	Mean		Median (IQR)
		Mean ± sd	(95% CI)	
Age (years)		36.4 ± 11.7	(35.8 to 37.0)	37.0 (18.0)
Marital status				
Single	620 (44.2)			
Married	642 (45.7)			
Separated	16 (1.1)			
Divorced	108 (7.7)			
Widower	9 (0.6)			
Living status during confinement				
In social isolation at home, not working and alone	40 (2.8)			
In social isolation at home, not working, with other people	472 (33.6)			
Working out in full-time	136 (9.7)			
Working out in part-time	104 (7.4)			
Teleworking at home, alone	72 (5.1)			
Teleworking at home, with other people	575 (41)			
Home quarantine	5 (0.4)			
Academic level				
Elementary	50 (3.6)			
Secondary	263 (18.7)			
Professional	107 (7.6)			
Superior	984 (70.1)			
Usual sleep duration				
Yes	856 (61.0)			
No	548 (39.0)			
Sleep quality satisfaction				
Yes	826 (58.8)			
No	578 (41.2)			
Higher food frequency				
Yes	634 (45.2)			
No	770 (54.8)			
Higher food quantity				
Yes	444 (31.6)			
No	960 (68.4)			
Careful food selection				
Yes	588 (41.9)			
No	816 (58.1)			
Time spent watching, reading or listening to the news about Coronavirus				
None	10 (0.7)			
Less than 1 h	517 (36.8)			
Between 1 to 3 h	744 (53.0)			
More than 3 h	129 (9.2)			
Another option	4 (0.3)			
Physical activity category				
Low	447 (31.8)			
Moderate	697 (49.6)			
High	260 (18.5)			
Total energy expenditure (METS)		1843 ± 2155	(1730 to 1956)	1206 (1942)
Anxiety state		45.1 ± 11.2	(44.5 to 45.7)	44.0 (15.0)
Anxiety trait		37.9 ± 10.3	(37.4 to 38.4)	36.0 (13.0)
Autonomy		4.43 ± 0.67	(4.40 to 4.47)	4.43 (0.86)
Competence		5.01 ± 0.88	(4.97 to 5.06)	5.00 (1.13)
Relatedness		4.95 ± 0.58	(4.92 to 4.98)	5.00 (0.88)

Notes: sd, standard deviation; 95% CI, confidence interval 95%; IQR, interquartile range.

**Table 2:** Gender-related distribution according to sample characteristics (*n* = 1403).

	Female	Male	*P*	Effect Size
	*n* = 977	*n* = 426		
				
Usual sleep duration [*n* (%)]			0.469	0.019
Yes	590 (60.4)	266 (62.4)		
No	387 (39.6)	160 (37.6)		
Sleep quality satisfaction [*n* (%)]			**<0.001**	0.127
Yes	535 (54.8)	291 (68.3)		
No	442 (45.2)	135 (31.7)		
Higher food frequency [*n* (%)]			**0.018**	−0.063
Yes	461 (47.2)	172 (40.4)		
No	516 (52.8)	254 (59.6)		
Higher food quantity [*n* (%)]			**0.001**	−0.085
Yes	334 (34.2)	109 (25.6)		
No	643 (65.8)	317 (74.4)		
Careful food selection [*n* (%)]			0.520	0.017
Yes	404 (41.4)	184 (43.2)		
No	573 (58.6)	242 (56.8)		
Time spent watching, reading or listening to news about Coronavirus [*n* (%)]			0.233	0.063
None	8 (0.8)	2 (0.5)		
Less than 1 h	374 (38.3)	143 (33.6)		
Between 1 to 3 h	502 (51.4)	241 (56.6)		
More than 3 h	89 (9.1)	40 (9.4)		
Another	4 (0.4)	0 (0)		
Total energy expenditure – METS (mean ± sd)	1780 ± 2188	1989 ± 2076	**0.024**	0.004
Anxiety state (mean ± sd)	46.7 ± 11.1	41.5 ± 10.4	**<0.001**	0.044
Anxiety trait (mean ± sd)	39.0 ± 10.3	35.3 ± 9.7	**<0.001**	0.030
Autonomy (mean ± sd)	4.42 ± 0.68	4.47 ± 0.66	0.628	0.000
Competence (mean ± sd)	4.97 ± 0.88	5.11 ± 0.86	**0.009**	0.005
Relatedness (mean ± sd)	4.96 ± 0.59	4.93 ± 0.55	0.383	0.001

Notes: sd, standard deviation; effect size was computed as *Phi* (ϕ) for categorical variables and as $r^{2}=\eta^{2}$ for continuous variables. Significant values are highlighted in bold.

**Table 3:** Age-related distribution according to sample characteristics (*n* = 1404).

	18–34	35–44	45–54	55–64	>65	*P*	Effect Size
	*n* = 607	*n* = 457	*n* = 254	*n* = 67	*n* = 19		
							
Usual sleep duration [*n* (%)]						**<0.001**	0.175
Yes	314 (51.7)	301 (65.9)	176 (69.3)	52 (77.6)	13 (68.4)		
No	293 (48.3)	156 (34.1)	78 (30.7)	15 (22.4)	6 (31.6)		
Sleep quality satisfaction [*n* (%)]						0.287	0.060
Yes	349 (57.5)	273 (59.7)	146 (57.5)	43 (64.2)	15 (78.9)		
No	258 (42.5)	184 (40.3)	108 (42.5)	24 (35.8)	4 (21.1)		
Higher food frequency [*n* (%)]						0.094	0.075
Yes	287 (47.3)	212 (46.4)	107 (42.1)	22 (32.8)	6 (31.6)		
No	320 (52.7)	245 (53.6)	147 (57.9)	45 (67.2)	13 (68.4)		
Higher food quantity [*n* (%)]						0.112	0.073
Yes	187 (30.8)	157 (34.4)	83 (32.7)	13 (19.4)	4 (21.1)		
No	420 (69.2)	300 (65.6)	171 (67.3)	54 (80.6)	15 (78.9)		
Careful food selection [*n* (%)]						**0.002**	0.112
Yes	220 (36.2)	198 (43.3)	128 (50.4)	33 (49.3)	9 (47.4)		
No	387 (63.8)	259 (56.7)	126 (49.6)	34 (50.7)	10 (52.6)		
Time spent watching, reading or listening to news about Coronavirus [*n* (%)]						**<0.001**	0.126
None	4 (0.7)	5 (1.1)	1 (0.4)	0 (0)	0 (0)		
Less than 1 h	257 (42.3)	178 (38.9)	59 (23.2)	18 (26.9)	5 (26.3)		
Between 1 to 3 h	313 (51.6)	235 (51.4)	152 (59.8)	38 (56.7)	6 (31.6)		
More than 3 h	33 (5.4)	37 (8.1)	41 (16.1)	11 (16.4)	7 (36.8)		
Another option	0 (0)	2 (0.4)	1 (0.4)	0 (0)	1 (5.3)		
Total energy expenditure—METS (mean ± sd)	1965 ± 2072^b^	1636 ± 2020 ^a^	1810 ± 2237	2094 ± 2647	2448 ± 3435	**0.008**	0.007
Anxiety state (mean ± sd)	45.4 ± 11.3	44.5 ± 10.9	45.4 ± 11.3	44.7 ± 11.9	44.6 ± 9.2	0.693	−0.001
Anxiety trait (mean ± sd)	40.1 ± 10.9 ^b,c,d,e^	36.3 ± 9.7 ^a^	36.3 ± 9.3 ^a^	36.0 ± 8.4 ^a^	34.5 ± 6.8 ^a^	**<0.001**	0.029
Autonomy (mean ± sd)	4.43 ± 0.69	4.44 ± 0.64	4.40 ± 0.6	4.48 ± 0.71	4.83 ± 0.61	0.104	0.003
Competence (mean ± sd)	4.83 ± 0.93 ^b,c,d^	5.14 ± 0.80 ^a^	5.18 ± 0.84 ^a^	5.13 ± 0.76 ^a^	5.16 ± 0.77	**<0.001**	0.026
Relatedness (mean ± sd)	4.98 ± 0.59	4.94 ± 0.56	4.91 ± 0.57	4.94 ± 0.63	5.05 ± 0.58	0.383	0.000

Notes: sd, standard deviation; ^a^—significant differences with the 18–34 age group; ^b^—significant differences with the 35–44 age group; ^c^—significant differences with the 45–54 age group; ^d^—significant differences with the 55–64 age group; ^e^—significant differences with the >65 age group; effect size was computed as *Phi* (ϕ) for categorical variables and as $\eta_{H}^{2}$ for continuous variables. Significant values are highlighted in bold.
